# Photoperiod Stress in *Arabidopsis thaliana* Induces a Transcriptional Response Resembling That of Pathogen Infection

**DOI:** 10.3389/fpls.2022.838284

**Published:** 2022-05-12

**Authors:** Anne Cortleven, Venja M. Roeber, Manuel Frank, Jonas Bertels, Vivien Lortzing, Gerrit T. S. Beemster, Thomas Schmülling

**Affiliations:** ^1^Dahlem Centre of Plant Sciences, Institute of Biology/Applied Genetics, Freie Universität Berlin, Berlin, Germany; ^2^Department of Molecular Biology and Genetics, Aarhus University, Aarhus, Denmark; ^3^Laboratory for Integrated Molecular Plant Physiology, Department of Biology, University of Antwerp, Antwerp, Belgium; ^4^Institute of Biology/Applied Zoology—Animal Ecology, Dahlem Centre of Plant Sciences, Freie Universität Berlin, Berlin, Germany

**Keywords:** photoperiod stress, oxidative stress, pathogen infection, plant defense response, *Arabidopsis*

## Abstract

Plants are exposed to regular diurnal rhythms of light and dark. Changes in the photoperiod by the prolongation of the light period cause photoperiod stress in short day-adapted *Arabidopsis thaliana*. Here, we report on the transcriptional response to photoperiod stress of wild-type *A. thaliana* and photoperiod stress-sensitive cytokinin signaling and clock mutants and identify a core set of photoperiod stress-responsive genes. Photoperiod stress caused altered expression of numerous reactive oxygen species (ROS)-related genes. Photoperiod stress-sensitive mutants displayed similar, but stronger transcriptomic changes than wild-type plants. The alterations showed a strong overlap with those occurring in response to ozone stress, pathogen attack and flagellin peptide (flg22)-induced PAMP triggered immunity (PTI), which have in common the induction of an apoplastic oxidative burst. Interestingly, photoperiod stress triggers transcriptional changes in jasmonic acid (JA) and salicylic acid (SA) biosynthesis and signaling and results in increased JA, SA and camalexin levels. These responses are typically observed after pathogen infections. Consequently, photoperiod stress increased the resistance of *Arabidopsis* plants to a subsequent infection by *Pseudomonas syringae* pv. *tomato* DC3000. In summary, we show that photoperiod stress causes transcriptional reprogramming resembling plant pathogen defense responses and induces systemic acquired resistance (SAR) in the absence of a pathogen.

## Introduction

As the earth turns around its own axis, the daily change of day and night results in the adaptation of life processes to this rhythm. The photoperiod is the duration of the light period during a day-night cycle of 24 h. Consequently, numerous developmental processes are controlled by the photoperiod (Jackson, 2009). Recently, it has been described that changes of the photoperiod, in particular a prolongation of the light period, provokes a stress response in *A. thaliana*. This identified form of abiotic stress is named photoperiod stress—originally circadian stress—(Nitschke et al., 2016, 2017; Roeber et al., 2022) and was first detected in cytokinin (CK)-deficient *Arabidopsis* plants as these plants are particularly photoperiod stress-sensitive. The photoperiod stress response starts during the night following a prolonged light period with a strong induction of stress marker genes such as *ZINC FINGER OF ARABIDOPSIS THALIANA12* (*ZAT12*) and *BON ASSOCIATED PROTEIN1* (*BAP1*), an increase in oxidative stress and the jasmonic acid (JA) concentration. The next day, the photosynthetic efficiency is strongly reduced in plant leaves and programmed cell death follows in strongly stressed and photoperiod stress-sensitive plants. A weaker molecular response was detected in wild-type (WT) *A. thaliana* (Nitschke et al., 2016; Abuelsoud et al., 2020). The nightly increase in oxidative stress coincides with a strong decrease in ascorbic acid redox state and an increased formation of peroxides (including H_2_O_2_), which is associated with a strong increase of *PEROXIDASE* (*PRX*) gene expression, enhanced PRX and decreased catalase activity. These stress symptoms are even more pronounced in photoperiod stress-sensitive mutants (Abuelsoud et al., 2020). CK, especially root-derived *trans*-zeatin derivatives (Frank et al., 2020), have a protective function, acting through the ARABIDOPSIS HISTIDINE KINASE 3 (AHK3) receptor and the transcriptional regulators ARABIDOPSIS RESPONSE REGULATOR2 (ARR2), ARR10 and ARR12. Similar protective functions of CK have been observed in response to other stresses (Pavlů et al., 2018; Cortleven et al., 2019). In addition, certain clock mutants of both the morning and evening loops (e.g., *cca1 lhy*, *elf3*) are photoperiod stress-sensitive (Nitschke et al., 2016). Common to photoperiod stress-sensitive clock mutants and CK-deficient mutant plants was a lowered expression or impaired function of CIRCADIAN CLOCK ASSOCIATED1 (CCA1) and LONG HYPOCOTYL (LHY), two key regulators of the circadian clock, which indicates that a functional clock is essential to cope with stress caused by altered light-dark rhythms (Nitschke et al., 2016). An impairment of CCA1 might result in increased oxidative stress as CCA1 is a key regulator of reactive oxygen species (ROS) homeostasis (Lai et al., 2012). An important function of the circadian clock is to anticipate the daily light-dark rhythm and to match the circadian clock with the environment. This is crucial for the regulation of numerous biological processes including the activity of plant hormones, the formation of and response to ROS and responses to plant pathogens (Covington et al., 2008; Harmer, 2009; Roden and Ingle, 2009; Seo and Mas, 2015; Carmela et al., 2018; Karapetyan and Dong, 2018).

Plant defense responses to pathogens involve a multilayered strategy including the primary innate immunity and a host-specific secondary immune response (Chisholm et al., 2006; de Wit, 2007). Pathogen-associated molecular patterns (PAMPs) are detected by pattern recognition receptors resulting in PAMP-triggered immunity (PTI). This primary innate immunity involves the induction of pathogen-responsive genes, ROS production or alterations in hormone signaling pathways involving salicylic acid (SA) and JA. Certain pathogens produce effector proteins that are encoded by avirulence genes to circumvent PTI. These pathogen-derived effectors can be counteracted by plant resistance proteins encoded by *R* genes resulting in effector-triggered immunity (ETI). Both PTI and ETI lead to similar plant responses and have comparable signaling pathways involving ENHANCED DISEASE SUSCEPTIBILITY1 (EDS1) and PHYTOALEXIN4 (PAD4), which promote *ISOCHORISMATE SYNTHASE1* (*ICS1*) expression and thus SA accumulation (Cui et al., 2017). Increased cellular SA levels activate several signaling cascades, among others, it induces biosynthesis of camalexin, which is the major phytoalexin formed during biotic stress responses (Glawischnig, 2007). EDS1 and PAD4 also regulate a SA-independent immunity pathway in which FLAVIN-DEPENDENT MONOOXYGENASE1 (FMO1) acts as an inducer of systemic acquired resistance (SAR) by regulating the production of *N*-hydroxypipecolic acid (NHP) (Bartsch et al., 2006; Hartmann et al., 2018).

Light is crucial to activate local and systemic plant resistance responses in response to pathogens (Ballaré, 2014; Roeber et al., 2021, 2022). Several studies have shown that in particular the length of the light period determines the strength of the plant immune response in *A. thaliana* (Cecchini et al., 2002; Griebel and Zeier, 2008). Cagnola et al. (2018) showed that transferring short day-grown *Arabidopsis* to long day photoperiod enhanced the resistance to the necrotrophic fungus *Botrytis cinerea* due to an improvement of the JA-related plant defense. Similarly, Shimizu et al. (2021) showed that plant resistance to the hemibiotrophic fungus *Pyricularia oryzae* (syn. *Magnaporthe oryzae*) was enhanced when a light period followed evening inoculations instead of the normal dark period. These studies indicate that the length of the light period within the photoperiod is crucial during plant pathogen defense responses. The length of the photoperiod also plays an important role in other stress responses, including the response to cold (Lee and Thomashow, 2012) and heat (Dickinson et al., 2018; Han et al., 2019).

In this study, we investigated transcriptional changes in response to photoperiod stress. We compared the transcriptomic landscape of WT plants and two photoperiod stress-sensitive mutants, *ahk2 ahk3* and *cca1 lhy*, in the course of the night following a prolonged light period (PLP). Photoperiod stress results in profound changes of transcript abundance with a distinct time-dependent profile. Among the differentially expressed genes (DEGs) responding to photoperiod stress are many ROS-responsive genes. Globally, the transcriptional changes caused by photoperiod stress resemble those induced by ozone stress, flagellin peptide (flg22)-induced PTI and pathogen attack, including deregulated expression of genes related to SA biosynthesis and signaling. Further, photoperiod stress increases the SA, JA, and camalexin concentrations resulting in enhanced resistance to a subsequent pathogen infection.

## Materials and Methods

### Plant Material and Growth Conditions

*Arabidopsis thaliana* accession Col-0 was used as WT. The following mutants were used: *ahk2-5 ahk3-7* (Riefler et al., 2006), *cca1-1 lhy-20* (Nitschke et al., 2016). *Arabidopsis* plants were grown on soil for 5 weeks under short day (SD) conditions (8 h light/16 h darkness) in a growth chamber with light intensities of 100–150 μmol m^–2^ s^–1^, using a combination of Philips Son-T Agros 400 W and Philips Master HPI-T Plus, 400 W/645 lamps, at 22°C and 60% relative humidity.

### Stress Treatment

**FIGURE 1:**
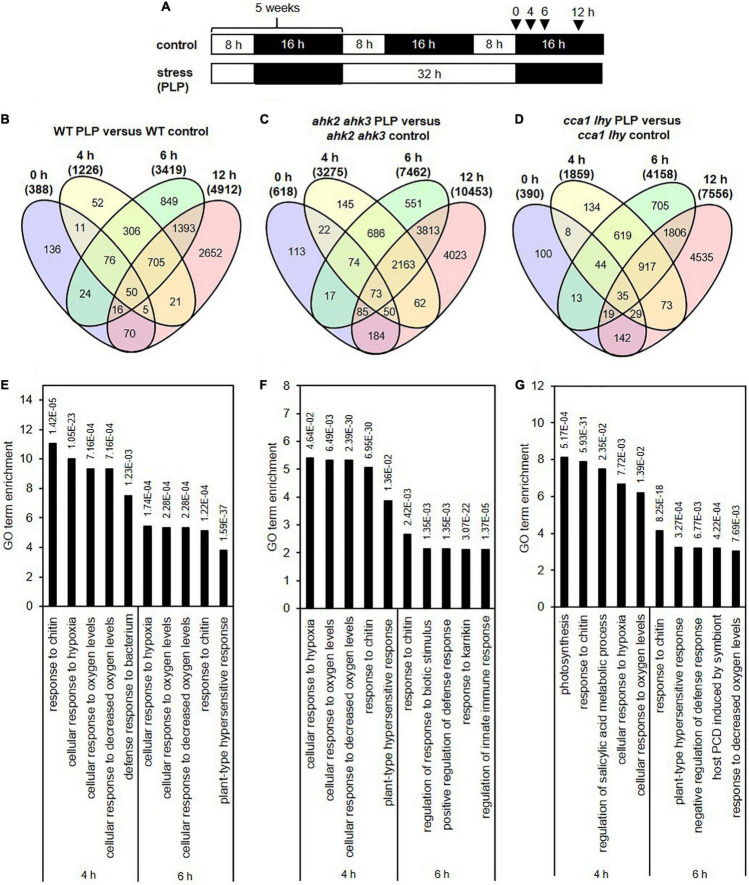
Significantly regulated genes in WT, *ahk2 ahk3* and *cca1 lhy* after photoperiod stress. **(A)** Experimental setup used in this study. 5-week-old short-day (SD)-grown plants were exposed to a prolonged light period (PLP) of 32 h followed by a normal SD night. White, light period; black, dark period. Arrows indicate sampling time points for RNA analysis. **(B–D)** Venn diagrams showing the overlap of DEGs at different time points for WT **(B)**, *ahk2 ahk3* **(C)** and *cca1 lhy* **(D)**. Numbers in brackets indicate the total number of DEGs (| fold-change| = 2; Bonferroni-corrected *p*-value ≤ 0.05) in PLP-treated plants compared with control plants at the different time points. **(E–G)** Top 5 GO enrichment terms for time point 4 and 6 h for WT **(E)**, *ahk2 ahk3* **(F)** and *cca1 lhy* **(G)**. A list of top-5 GO enrichment terms pro timepoint can be found in Supplementary Table 2. An overview of the gene regulation for the comparisons between PLP and control treatments for WT, *ahk2 ahk3* and *cca1 lhy* is shown for all time points in Supplementary Data 2. A core-set of photoperiod stress-responsive genes is listed in Supplementary Table 3 and the top 20 most highly regulated genes at each time point are listed in Supplementary Tables 6–11.

For stress treatments, 5-week-old SD-grown plants were used. Photoperiod stress was induced by a 24 h prolongation of the light period (prolonged light period, PLP) followed by a normal 16 h night (Figure 1A). Control plants remained under SD conditions. Stress parameters were analyzed in leaves 7–10. The harvest during the dark period was performed in green light.

### Plant Pathogen Infection

Leaves 11–13 of 4-week-old *Arabidopsis* plants were used for inoculation with *Pseudomonas syringae* pv. *tomato* DC3000 (*Pst*), the method was carried out as described by Griebel and Zeier (2008). For pathogen infection we used an initial inoculum at OD_600_ of 0.002.

### Analysis of Transcript Levels by RNA-Seq and Quantitative Real-Time PCR

Total RNA was extracted from leaf material (leaves 7–10). Only the distal parts of leaves 7–10 were harvested which is the most affected part of the leaves. Leaves were flash-frozen in liquid nitrogen and homogenized with a Retsch Mixer Mill MM2000 (Retsch, Haan, Germany). Total RNA was extracted using the NucleoSpin^®^ RNA plant kit (Machery and Nagel, Düren, Germany) as described in the user’s manual and in Frank et al. (2020). For RNA-seq analysis, RNA was isolated from three biological samples at four different time points. The isolated RNA was send to BGI (Hongkong, China) and processed as described in Cortleven et al. (2019). In brief, a NanoDrop NA-1000 and a Bioanalyzer Agilent 2100 (Agilent Technologies, Santa Clara, CA, United States) were used to check RNA concentration, integrity and rRNA contamination. After DNase I treatment, mRNA was enriched by using oligo (dT) magnetic beads and fragmented into shorter fragments. First-strand cDNA was synthesized by using random hexamer primers, followed by second strand synthesis. After purification, end repair, and 3′ end single-nucleotide A (adenine) addition, sequence adaptors were ligated. Following PCR amplification and quality control by the Agilent 2100 Bioanalyzer and ABI StepOnePlus Real-Time PCR System (Thermo Fischer Scientific, Waltham, MA, United States), the library products were sequenced on the BGI SEQ-500 platform. More than 22 million raw sequencing reads were obtained for each sample. After the removal of adaptors and low-quality reads, the obtained clean reads (approximately 21 million) were stored in FASTQ format (Cock et al., 2010). Sequences were aligned to the TAIR11 *Arabidopsis* reference genome using Bowtie2 (Langmead and Salzberg, 2012). Gene expression levels were quantified using RSEM (Li and Dewey, 2011) and DEGs were identified using the DESeq method (Love et al., 2014) considering three different parameters (time, genotype and treatment) with the following default criteria: fold change ≥ 2 and Bonferroni correction (*p*-value ≤ 0.05). RNA-seq data are deposited in NCBI’s Gene Expression Omnibus and are accessible through GEO Series accession number GSE173899.

Gene Ontology (GO) annotation was performed using PANTHER (Mi et al., 2013, 2019). Heatmaps were created using MEV (MultiExperiment Viewer; Saeed et al., 2003; Howe et al., 2011). For cluster analysis, MEV was used. Quality Threshold (QT) clustering was done using the following parameters: diameter: 0.7; minimum cluster size: 50; Euclidean distance or with a Pearson’s correlation: diameter: 0.3, minimum cluster size: 10. For hierarchical clustering, Euclidean distance and average linkage was used. PCA analysis was performed using the PCAplot function in R (version 3.6.2).

For quantitative real-time PCR (qRT-PCR), leaf material was collected at the same time points as for RNA-seq analysis. Quantitative real-time PCR analysis was performed as described in Cortleven et al. (2019). Sequences of primers used for gene expression analysis are listed in Supplementary Table 1. Gene expression data were normalized against three or four different nuclear-encoded reference genes (*UBC21*, *TAFII15*, *PP2A*, and/or *MCP2A*) according to Vandesompele et al. (2002) and expressed relative to the control treatment at time point 0 h which was set to 1.

### Comparison of Transcript Profiles of Biotic and Abiotic Stresses With the Photoperiod Stress Transcript Profile

The transcriptomic profile of photoperiod stress was compared with the transcriptomic profiles of different specific biotic and abiotic stresses. The percent overlap of changes caused by photoperiod stress with those caused by other stresses was calculated as follows:


(1) $$\frac{\begin{array}{c} n\,u\,m\,b\,e\,r\,p\,h\,o\,t\,o\,p\,e\,r\,i\,o\,d\,s\,t\,r\,e\,s\,s\,D\,E\,G\,s\,c\,o\,m\,m\,o\,n\\ w\,i\,t\,h\,t\,h\,e\,D\,E\,G\,s\,o\,f\,t\,h\,e\,s\,p\,e\,c\,i\,f\,i\,c\,s\,t\,r\,e\,s\,s \end{array}}{\begin{array}{c} t\,o\,t\,a\,l\,n\,u\,m\,b\,e\,r\,o\,f\,D\,E\,G\,s\,i\,d\,e\,n\,t\,i\,f\,i\,e\,d\,t\,o\,b\,e\,s\,p\,e\,c\,i\,f\,i\,c\\ f\,o\,r\,t\,h\,e\,b\,i\,o\,t\,i\,c\,o\,r\,a\,b\,i\,o\,t\,i\,c\,s\,t\,r\,e\,s\,s \end{array}}\times 100\%.$$

### Determination of Jasmonic Acid, Salicylic Acid, and Camalexin Levels

JA, SA and camalexin were extracted and their levels determined by UPLC-MS/MS (Q-ToF-ESI; Synapt G2-S HDMS; Waters^®^, Milford, Massachusetts, United States) as described in Valsamakis et al. (2020).

### Statistical Analysis

Statistical analyzes were performed using SAS v.9.2 (SAS Institute GmbH, Heidelberg, Germany) or R (version 3.6.2). Data were analyzed by a Welch *t*-test or ANOVA followed by Tukey’s *post-hoc* test. Normality and variance homogeneity of datasets were tested using the Shapiro-Wilk and Levene tests (Neter et al., 1996). To meet the assumptions, datasets were transformed using logarithmic or square root transformations.

## Results

### Characteristics of Transcriptomic Changes in Response to Photoperiod Stress

In order to obtain genome-wide information of the transcriptional response to photoperiod stress, we analyzed changes of transcript abundance during the night following a light period that was prolonged by 24 h in SD-grown plants (WT, *ahk2 ahk3, cca1lhy*). This treatment causes a strong stress response (Nitschke et al., 2016), but shorter prolongations of the photoperiod in the range of few hours also cause a significant although weaker stress response (Abuelsoud et al., 2020). Samples for RNA-seq analysis were harvested at different time points (0, 4, 6, and 12 h) following the prolonged light treatment (Figure 1A). These time points reflected the timing of stress responses occurring during the night with photoperiod stress marker gene activation (*BAP1* and *ZAT12*) starting around 5 h in WT and the photoperiod stress-sensitive mutants. Visible phenotypical consequences (flabby leaves in photoperiod stress-sensitive genotypes) appear about 8 h after the start of the night and coincide with a stronger induction of the photoperiod stress marker genes and the formation of ROS (Nitschke et al., 2016; Abuelsoud et al., 2020). Thus, the time points for sampling were chosen to allow monitoring of early transcriptional changes occurring before the onset of visible stress symptoms as well as to detect later transcriptional changes during the night when physiological and phenotypical consequences start to appear. A scheme of the experimental setup is shown in Figure 1A. Principal component analysis (PCA) of the DEGs showed that control samples cluster together (red circle; Supplementary Figure 1A) indicating that changes in gene expression due to the genotype are much smaller than those caused by the treatment. Samples harvested at the end of the PLP (0 h time point) cluster together with the control samples suggesting that control and photoperiod stress-treated samples do not differ significantly at timepoint 0 h. The 4 h and 6 h time points cluster separately from control samples and a division among the different genotypes is visible. Especially at the 12 h time point, a strong separation of the genotypes (blue circles; Supplementary Figure 1A) is evident. At this time point, the *ahk2 ahk3* and *cca1 lhy* samples are clearly divergent from WT, which is consistent with the stronger photoperiod stress phenotype of these mutants (Nitschke et al., 2016).

We analyzed how many genes were regulated dependent on the genotype, time and treatment component (Supplementary Figure 1B). In total 10,278 genes were differentially expressed in a genotype-dependent manner, 17,465 genes in a time-dependent manner and 16,612 genes in a treatment-dependent manner (across genotypes and time points). The genotype-dependent DEGs were analyzed in detail by clustering and GO term overrepresentation analysis. Quality Treshold (QT) clustering revealed 21 different clusters (Supplementary Figure 2). Fifty two percent of all DEGs are found in cluster 1 and 2 which showed a downregulation (cluster 1) or an upregulation (cluster 2) during the night following the photoperiod stress treatment. This regulation has a higher amplitude in the photoperiod stress-sensitive mutants (Supplementary Figures 1C,D). According to GO term analysis, genes involved in photosynthesis- or chloroplast-related processes are overrepresented in cluster 1, whereas genes involved in autophagy, responses to endoplasmatic reticulum stress and Golgi vesicle transport are overrepresented in cluster 2 (Supplementary Figures 1E,F).

To get more insight in the DEGs following photoperiod stress, we made pairwise comparisons between photoperiod stress-treated and control samples for each genotype and time point. In all genotypes, the number of DEGs increased over time (Figures 1B–D and Supplementary Datas 1, 2). For instance, in WT there are 388 DEGs at time point 0 h, 1,226 DEGs at time point 4 h, 3,419 DEGs at time point 6 h and 4,912 DEGs at time point 12 h (Figure 1B and Supplementary Datas 1, 2). A large number of the early-regulated genes showed also an increased steady state level at later time points. The number of DEGs increased over time in all different genotypes, but the number of regulated genes being considerable higher in the mutants than in WT, which reflects their increased photoperiod stress sensitivity. The total number of DEGs at time point 12 h is 10,453 in *ahk2 ahk3* and 7,556 in *cca1 lhy* reflecting their higher sensitivity to PLP.

GO enrichment analysis of the DEGs identified by comparing photoperiod stress-treated and control samples, revealed that cellular responses to oxygen levels, response to chitin and plant-type hypersensitive responses and positive regulation of defense responses were among the top five significantly enriched GO terms at 4 and 6 h in all genotypes (Figures 1E–G and Supplementary Table 2). This indicates that the early changes on the transcriptomic landscape of photoperiod stress can be associated with responses to oxygen and biotic stress responses, which have in common the occurrence of oxidative stress (Wojtaszek, 1997; Bolwell et al., 2002; Fukao and Bailey-Serres, 2004; Schmidt et al., 2018).

There are no indications for a photoperiod stress response at time point 0 h as neither photoperiod stress marker genes nor stress response genes are upregulated at this time point (Nitschke et al., 2016; Frank et al., 2020). Therefore, we considered the changes at the 0 h time point as genotype-dependent effect caused by the prolongation of the light period that do not belong to the specific photoperiod stress response occurring during the night. However, DEGs at time point 0 h might be relevant for the perception of photoperiod stress and the development of the initial response. All genotypes had 90 genes in common that were significantly affected by PLP at time point 0 h (Table 1 and Supplementary Table 3). GO term analysis revealed that this core-set of DEGs is related to the circadian clock.

**TABLE 1:** Selection of the core-set of photoperiod stress-responsive genes.

	WT (log_2_ FC)			*ahk2 ahk3* (log_2_ FC)			*cca1 lhy* (log_2_ FC)			
			
AGI	4 h	6 h	12 h	4 h	6 h	12 h	4 h	6 h	12 h	Short description
**Upregulated DEGs**										
AT3G46080	7.42	6.63	7.46	5.87	9.53	4.36	7.03	9.01	4.89	C2H2-type zinc finger family protein
AT2G45760	5.94	7.47	5.63	6.24	8.84	4.39	8.50	8.90	4.63	Encodes a protein that is similar to BONZAI1-binding protein BAP1 (BAP2)
AT4G01360	5.91	6.79	6.74	6.14	8.90	3.87	7.28	8.23	3.91	Encodes a protein related to BYPASS1 (BPS3)
AT1G07160	5.74	6.31	5.31	5.53	6.59	3.24	6.91	8.02	3.01	Protein phosphatase 2C family protein
AT4G08555	5.55	8.49	7.87	5.45	8.54	4.12	7.02	9.66	4.65	hypothetical protein
AT1G26380	5.52	6.97	8.19	5.22	7.44	4.43	6.74	9.00	4.28	FAD-LINKED OXIDOREDUCTASE 1 (FOX1)
AT5G66890	5.46	10.34	7.69	6.77	11.22	4.51	8.85	12.08	4.98	N REQUIREMENT GENE 1.3 (NRG1.3)
AT2G32210	5.42	6.70	6.05	5.87	7.62	3.85	6.29	7.87	4.24	CYSTEINE-RICH TRANSMEMBRANE MODULE 6 (ATHCYSTM6)
AT5G64870	5.37	5.72	4.61	6.17	7.93	3.18	6.44	7.08	3.03	FLOTILLIN3 (FLOT3)
AT1G18300	5.36	5.52	3.96	4.59	5.14	3.21	7.26	7.37	3.03	NUDIX HYDROLASE HOMOLOG 4 (NUDT4)
AT2G27080	5.26	4.88	3.22	4.23	5.03	3.06	6.34	6.52	2.63	NDR/HIN1-LIKE 13 (NHL13)
AT1G19020	5.21	6.52	6.32	5.89	6.84	3.50	6.84	7.87	3.90	HYPOXIA RESPONSE UNKNOWN PROTEIN 35 (HUP35)
AT4G37290	5.20	5.32	8.32	5.61	6.42	4.10	6.42	8.14	5.35	PRECURSOR OF PAMP-INDUCED PEPTIDE 2 (PREPIP2)
AT5G59820	5.20	6.55	5.86	4.13	4.78	3.29	6.03	7.39	4.45	RESPONSIVE TO HIGH LIGHT 41 (RHL41; ZAT12)
AT1G07135	5.03	5.62	4.45	5.53	5.22	2.75	6.34	6.34	2.77	Glycine-rich protein
AT3G28340	4.95	5.43	4.47	5.03	5.52	3.06	6.24	6.44	3.25	GALACTURONOSYLTRANSFERASE-LIKE 10 (GATL10)
AT2G32140	4.93	5.98	5.40	5.68	7.05	3.90	5.83	7.19	4.12	Transmembrane receptor
AT5G64310	4.87	5.83	4.28	4.62	6.73	3.43	5.77	6.33	3.28	ARABINOGALACTAN PROTEIN 1 (AGP1)
AT5G41750	4.85	5.85	4.30	5.63	6.71	2.62	5.67	6.39	2.68	Disease resistance protein (TIR-NBS-LRR class) family
AT2G32190	4.85	6.33	6.35	5.23	8.20	3.56	6.27	7.92	4.57	CYSTEINE-RICH TRANSMEMBRANE MODULE 4 (ATHCYSTM4)
**Downregulated DEGs**										
AT1G11130	–1.03	–1.56	–2.78	–1.11	–1.94	–3.65	–1.64	–2.48	–6.30	STRUBBELIG-RECEPTOR FAMILY 9 (SRF9);SCRAMBLED (SCM)
AT1G27360	–1.12	–1.48	–1.87	–1.15	–1.82	–2.11	–1.48	–2.37	–2.31	SQUAMOSA PROMOTER-LIKE 11 (SPL11)
AT4G07825	–1.13	–1.78	–1.34	–1.41	–1.55	–1.40	–1.86	–2.37	–2.02	Transmembrane protein
AT3G18320	–1.14	–1.90	–2.10	–1.77	–2.24	–1.84	–2.32	–3.15	–2.49	F-box and associated interaction domains-containing protein
AT3G52170	–1.28	–1.63	–1.69	–1.12	–1.36	–1.66	–1.30	–2.08	–2.69	DNA binding protein
AT2G05995	–1.47	–2.04	–3.93	–2.12	–1.69	–3.71	–2.24	–2.65	–4.38	Other_RNA

*Fold changes are sorted according to the 4 h time point in WT. Only statistically significant differently regulated genes are shown (Bonferroni < 0.05). FC, fold change.*

*The complete core-set of photoperiod stress-responsive genes are listed in Supplementary Table 3.*

The DEGs that are shared between WT, *ahk2 ahk3* and *cca1 lhy* at time points 4, 6, and 12 h resulted in a core-set of 388 photoperiod stress-regulated genes, of which 382 genes were upregulated and 6 downregulated (Table 1 and Supplementary Table 3). GO overrepresentation analysis (Supplementary Table 4) revealed that the core-set of photoperiod stress-regulated genes belong mostly to cellular responses to hypoxia or to oxygen levels and defense responses to pathogens. Hence, the results support that photoperiod and oxidative stress are similar. We further evaluated these similarities below.

**FIGURE 2:**
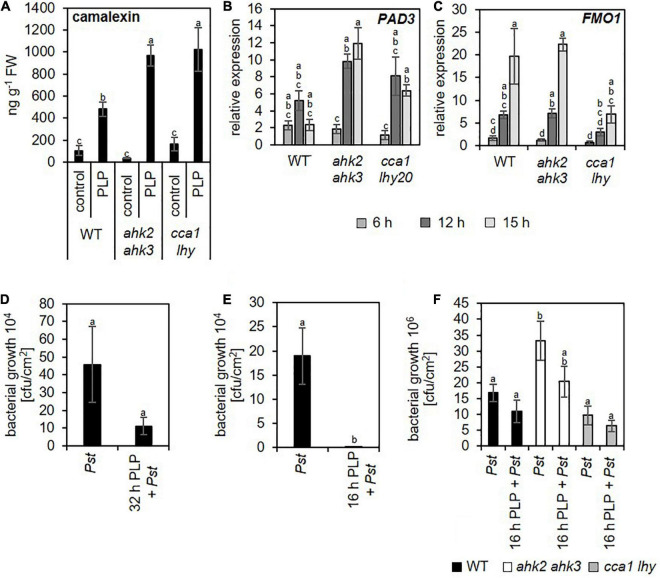
Overlap of DEGs of the different genotypes in response to photoperiod stress. Venn diagrams showing the overlap of DEGs between WT, *ahk2 ahk3* and *cca1 lhy* at different time points after photoperiod stress treatment. 5-week-old short-day (SD)-grown plants were exposed to an extended light period of 32 h followed by a normal SD night (see Figure 1A). Numbers in brackets indicate the total number of DEGs (| fold-change| = 2; Bonferroni-corrected *p*-value ≤ 0.05) in prolonged light period (PLP)-treated plants compared to control plants at the respective time points. The bold red number in the Venn diagram indicates the number of DEGs occurring specifically in the stress-sensitive genotypes *ahk2 ahk3* and *cca1 lhy.*

The transcriptional changes in the two stress-sensitive genotypes showed, beside a larger number of DEGs, a number of peculiarities which might be functionally relevant for the enhanced phenotype and which will be explored in more detail elsewhere. About two third of the responsive genes of WT were also found in the two photoperiod stress-sensitive genotypes. However, during the course of the night, there was an increasing number of DEGs characteristic for the stress-sensitive genotypes *ahk2 ahk3* and *cca1 lhy* (Figure 2 and Supplementary Table 5), which do not occur in WT. One obvious difference to WT was the downregulation of numerous *SMALL AUXIN UP-RNA* (*SAUR*) genes. Photoperiod stress is characterized by an oxidative burst (Abuelsoud et al., 2020) which is more pronounced in the photoperiod stress-sensitive genotypes. Oxidative stress is known to affect auxin signaling (Blomster et al., 2011) which might be a cause for the downregulation of the numerous *SAUR* genes in these genotypes. Investigation of the functional relevance of these *SAUR* genes is an interesting direction for future research on the photoperiod stress syndrome.

### Numerous Genes Related to Oxidative Stress Are Responsive to Photoperiod Stress

**FIGURE 3:**
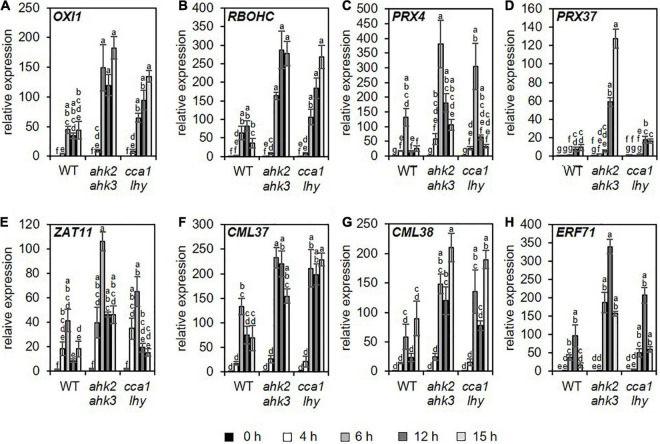
Genes related to oxidative stress are upregulated in all genotypes in response to photoperiod stress. Plants were grown under short day conditions for 5 weeks before exposure to a 32 h prolonged light period (PLP) (see Figure 1A). **(A–H)** Relative expression of *OXI1*, *RBOHC, PRX4, PRX37, ZAT11, CML37, CML28*, and *ERF71* in WT, *ahk2 ahk3* and *cca1 lhy* plants at different time points during the night following PLP measured by qRT-PCR. Only results for the response to PLP-treatment are shown. An overview of all expression levels including control conditions is shown in Supplementary Table 12. All values are expressed relative to WT control at 0 h, which was set to 1. Error bars represent SE (*n* ≥ 3). Letters indicate different statistical groups (*p* ≤ 0.05) as determined by ANOVA followed by Tukey’s *post-hoc* test.

A previous study (Abuelsoud et al., 2020) demonstrated that photoperiod stress is associated with a nightly increase in peroxide content resulting in an oxidative burst due to increased peroxidase and decreased catalase activity. Therefore, we focused especially on changed transcript levels of genes related to oxidative stress. Consistent with the increased oxidative stress after photoperiod stress (Abuelsoud et al., 2020), genes related to oxidative stress were found among the top 20 strongest up- and downregulated DEGs for the different genotypes (Supplementary Tables 6–11). Among them are *OXIDATIVE SIGNAL-INDUCIBLE1* (*OXI1*), *RESPIRATORY BURST OXIDASE HOMOLOG C* (*RBOHC*), *PEROXIDASE4* (*PRX4)*, *PRX37*, *ZAT11*, *CALMODULIN LIKE 37* (*CML37*), *CML38*, and *ETHYLENE RESPONSE FACTOR 71* (*ERF71*). *OXI1* encodes a protein kinase necessary for oxidative burst-mediated signaling in *Arabidopsis* (Rentel et al., 2004). *RBOHC* is required for the production of ROS in response to an extracellular ATP stimulus (Kaya et al., 2019) and both *PRX4* and *PRX37* encode apoplastic oxidative burst peroxidases (Valerio et al., 2004; Daudi et al., 2012; O’Brien et al., 2012). The TFs, ZAT11, and ERF71, are involved in nickel ion tolerance (Liu et al., 2014) or hypoxia (Hess et al., 2011), respectively, and can be induced by H_2_O_2_ (Hieno et al., 2019). The calmodulin-like proteins CML37 and CML38 are involved in drought stress and herbivore tolerance (Scholz et al., 2014, 2015) or in hypoxia stress (Lokdarshi et al., 2016), respectively. The induction of these genes was confirmed for all three genotypes by qRT-PCR analysis, which also showed that the induction was stronger in the mutants (Figure 3 and Supplementary Table 12).

**FIGURE 4:**
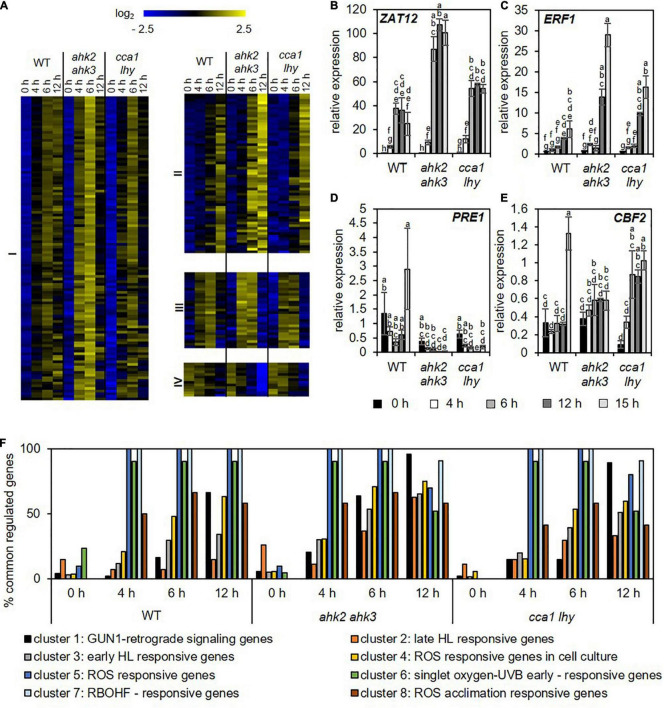
Photoperiod stress is associated with a strong transcriptional regulation of genes involved in oxidative stress. **(A)** QT clustering of log_2_ fold change to corresponding control of genes encoding TFs identified by Gadjev et al. (2006), Lai et al. (2012), Hieno et al. (2019) and Zandalinas et al. (2019), and as ROS-responsive genes. The overlap of the genes identified in the different studies is shown in Supplementary Figure 3. Four different clusters (I–IV) were identified. An overview of the regulation of these ROS responsive genes after exposure to a prolonged light period (PLP) are provided in Supplementary Data 3. **(B–E)** Relative expression of representative genes of each of the four cluster shown in **(A)**, i.e., *ZAT12* **(B)**, *ERF1* **(C)**, *PRE1* **(D)**, and *CBF2* **(E)** in WT, *ahk2 ahk3* and *cca1 lhy* plants during the night following the 32 h PLP measured by qRT-PCR. Only results for PLP-treatment are shown. An overview of all expression levels including control conditions are provided in Supplementary Table 12. All values are expressed relative to 0 h WT control which was set to 1. Error bars represent SE (*n* ≥ 3). Letters indicate different statistical groups as determined by ANOVA followed by Tukey’s *post-hoc* test. **(F)** Percentage of photoperiod stress-responsive genes that are co-regulated with the genes of the ROS wheel as defined by Willems et al. (2016). An overview of the regulation of these transcriptome profiles after photoperiod stress is given in Supplementary Data 5.

To investigate the response profile of the oxidative stress-regulated genes during photoperiod stress, we used datasets of several studies identifying core-sets of ROS-responsive genes for comparisons to our dataset. Hieno et al. (2019) unraveled a core-set of 60 H_2_O_2_-responsive transcription factors (TFs) after H_2_O_2_ treatment; Zandalinas et al. (2019) identified in response to short high light treatment 82 H_2_O_2_- and RBOHD-dependent genes and Lai et al. (2012) investigated the relation between the circadian clock and ROS-responsive genes and identified a core-set of 167 genes of which 140 were clock-regulated. In addition, transcriptional profiles specific for the response to H_2_O_2_, superoxide and singlet oxygen were identified by Gadjev et al. (2006). Overall, these four different core-sets of ROS-responsive genes showed only a small overlap (Supplementary Figure 3A), probably due to the different experimental setups used to increase ROS production. Therefore, these sets of genes were pooled to form a new master core-set of 283 ROS-responsive genes (Supplementary Data 3). QT clustering of transcript levels after photoperiod stress of this master core-set of ROS-responsive genes indicated that there is a strong regulation of these genes starting at 4 h during the dark relaxation (Figure 4A). Four different clusters were identified: Cluster I shows time-dependent upregulation of genes starting at the 4 h time point; Cluster II shows time-dependent upregulation of genes starting at the 6 h time point; Cluster III shows first upregulation of genes after 4 h and 6 h and then a decrease in expression at the 12 h time point; cluster IV shows time-dependent downregulation of genes. In all clusters, the response of the photoperiod stress-sensitive mutants *ahk2 ahk3* and *cca1 lhy* is stronger. Transcript levels of representative genes (*ZAT12*, *ERF1*, *PACLOBUTRAZOL RESISTANCE1* (*PRE1*) and *C-REPEAT/DRE BINDING FACTOR2* (*CBF2*) of the different clusters measured by qRT-PCR confirm the transcriptional regulation (Figures 4B–E). In addition, we analyzed the proportion of genes of the ROS core-set that are regulated at different time points in the different genotypes after photoperiod stress. Results showed that in all genotypes this proportion increased over time with the highest co-regulation at time points 6 and 12 h (Supplementary Figure 3B).

Comparison of the photoperiod stress-responsive transcriptomic profile with the distinct superoxide-, singlet oxygen- and H_2_O_2_-induced gene profiles identified by Gadjev et al. (2006) (Supplementary Figure 3C) revealed a strong overlap with the singlet oxygen-induced transcript profile (Supplementary Figure 3B and Supplementary Data 4). Similarly, a strong overlap with singlet oxygen-UV-B early, RBOHF-related and oxidative stress (ROS)-related transcript profiles (Figure 4F and Supplementary Data 5) can be recognized when comparing the core-set of DEGs after photoperiod stress with the different ROS footprints (Willems et al., 2016). Together, these results indicate the involvement of ROS signaling on the transcriptomic response to photoperiod stress.

Because photoperiod stress causes an oxidative burst (Abuelsoud et al., 2020), we investigated the transcriptional regulation of 221 genes encoding enzymes involved in the scavenging of ROS. QT cluster analysis revealed three major clusters (Supplementary Figure 4 and Supplementary Data 6). Cluster I showed an upregulation of genes over time in all genotypes, which is stronger in the photoperiod stress-sensitive genotypes, including the photoperiod stress-responsive *PRX34* (Abuelsoud et al., 2020), Cluster II shows a downregulation of genes over time, which is even stronger in the stress-sensitive mutants. *CAT2* (*CATALASE2*) is one of the genes of cluster 2. This downregulation of *CAT2* is in agreement with Abuelsoud et al. (2020) who found a decreased catalase activity after photoperiod stress. Cluster III consists of genes with a particular high expression at 4 h and 6 h time points for *ahk2 ahk3* mutants. *PRX4*, which was identified by Abuelsoud et al. (2020) as one of the strongly regulated genes upon photoperiod stress in relation to oxidative stress, belongs to this cluster. Among the top 20 significantly regulated genes are also a number of genes encoding glutathione-S-transferases (GSTs) (Supplementary Tables 6–11). GSTs are involved in the metabolization of toxic reactive compounds such as ROS during an oxidative burst (Gallé et al., 2019).

Taken together, these results indicate that photoperiod stress affects the expression of genes encoding enzymes involved in the scavenging of oxidative stress.

### The Transcriptional Changes to Photoperiod Stress Resemble Transcriptional Changes Caused by Pathogen Attack and Ozone Stress

**FIGURE 5:**
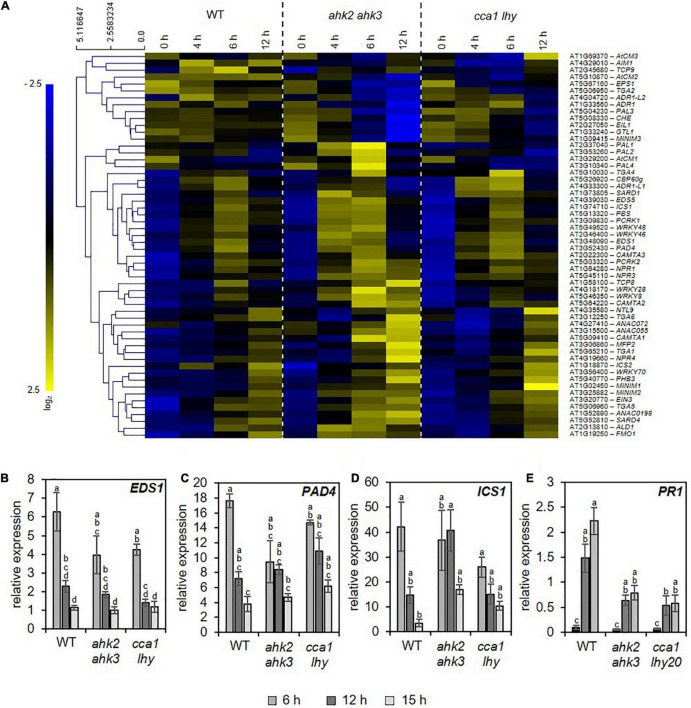
Photoperiod stress transcript profiles are similar to transcript profiles in response to pathogen and ozone stress. Percentage of genes commonly regulated in response to photoperiod stress and various abiotic and biotic stresses. An overview of the responses of the transcripts altered by these different stresses to photoperiod stress is provided in Supplementary Data 7. HL, high light; BL, blue light.

Photoperiod stress is a relatively new form of abiotic stress and not much is known about similarities with other stresses. We therefore compared the transcriptomic profile of plants in response to photoperiod stress with those in response to other biotic (Truman et al., 2007; Winkelmüller et al., 2021) and abiotic stresses, including a shift to blue light (BL), drought stress, heat stress, cold stress, salt stress, ozone treatment, fluctuating light and high light stress (Lee et al., 2005; Tosti et al., 2006; Kleine et al., 2007; Truman et al., 2007; Huang et al., 2008, 2019; Larkindale and Vierling, 2008; Ding et al., 2014; Schneider et al., 2019; Figure 5 and Supplementary Data 7). In addition, we compared our dataset with the transcriptomic profile of circadian clock-regulated genes (Covington et al., 2008) as a previous study found a link between photoperiod stress and the circadian clock (Nitschke et al., 2016). At the 0 h time point, the number of commonly regulated genes was relatively low in all genotypes, which indicate that the photoperiod stress response starts later during the night following the PLP, similar to the ROS-responsive transcript profile overlaps (Figure 4). Ozone treatment, flg22-induced PTI and pathogen attack showed the highest number of commonly regulated genes with photoperiod stress treatment (Figure 5). Already after 4 h, approximately 37% of photoperiod stress-regulated genes were identical to those regulated by ozone stress. The percentage of commonly regulated genes even increased to almost 70% at time point 12 h. 50% of the genes responding to pathogen attack and approximately 70% of the genes responding to flg22-induced PTI were also regulated by photoperiod stress at time point 12 h. In the photoperiod stress-sensitive mutants, the overlap between the transcriptional response to photoperiod stress and other stresses was stronger, however, again the highest overlap was detected between photoperiod and ozone stress, pathogen attack and flg22-induced PTI. Interestingly, the stresses that show the highest overlap of their DEGs with photoperiod stress cause an oxidative burst as does photoperiod stress (Abuelsoud et al., 2020).

Conspicuously, the transcriptomic response to photoperiod stress shows overlap with the response to high light (HL) stress (Figure 5). However, the photoperiod stress response differs from a HL stress response as plants display an effect directly after the HL stress, e.g., reduced photosynthetic capacity, while after photoperiod stress treatment plants only show a stress phenotype during the following night (Cortleven et al., 2014). Moreover, a prolongation of the light period alone is not causative for the photoperiod stress response as a night of at least 7.5 h is necessary to induce a cell death phenotype specific for the photoperiod stress response (Nitschke et al., 2016).

### Photoperiod Stress Induces Pathogen Defense Responses

**FIGURE 6:**
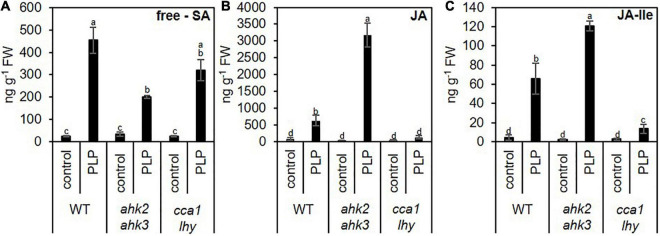
Photoperiod stress results in transcriptional regulation of salicylic acid biosynthesis and signaling. **(A)** Hierarchical clustering of genes involved in salicylic acid (SA) biosynthesis and signaling (Pearson’s correlation). An overview of the regulation of these signaling genes after photoperiod stress treatment is provided in Supplementary Data 8. **(B–E)** Relative expression of *EDS1* **(B)**, *PAD4* **(C)**, *ICS1* **(D)** and *PR1* **(E)** in WT, *ahk2 ahk3* and *cca1 lhy* plants during the night following the 32 h prolonged light period (PLP) measured by qRT-PCR. All values are expressed relative to 0 h WT control which was set to 1. Error bars represent SE (*n* ≥ 3). Letters indicate different statistical groups (*p* ≤ 0.05) determined by ANOVA followed by Tukey’s *post-hoc* test.

**FIGURE 7:**
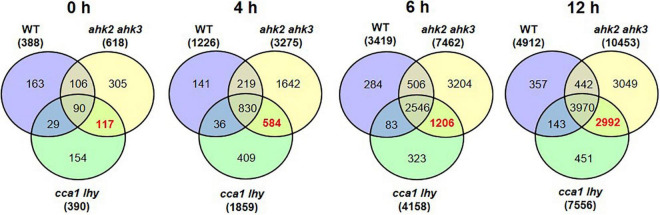
Photoperiod stress alters the levels of phytohormones involved in plant pathogen defense. Five-week-old SD-grown plants (WT, *ahk2 ahk3* and *cca1 lhy*) were exposed to an extended light period of 32 h followed by a normal SD night. **(A)** Salicylic acid (SA), **(B)** jasmonic acid (JA) and **(C)** jasmonic acid-isoleucine (JA- Ile) were measured at 15 h after the end of the prolonged light period treatment. Error bars represent SE (*n* ≥ 7). Letters indicate different statistical groups (*p* ≤ 0.05) as determined by a multiple Welch *t*-test with FDR correction.

The similarity of the transcriptional responses to photoperiod stress, pathogen attack and ozone stress (Figure 5) motivated us to explore possible links between these response pathways. Besides the oxidative burst evoked by these stresses, JA and SA biosynthesis and signaling are common signaling pathways affected by these stresses. Both, JA and SA, are involved in downstream signaling of PTI and ETI (Zhang et al., 2018). During ozone stress, SA accumulates (Yalpani et al., 1994; Sharma et al., 1996) and the ozone-induced hypersensitive cell death is modulated by JA signaling (Rao et al., 2000). Therefore, we explored the expression pattern of SA (Figure 6A and Supplementary Data 8) and JA (Supplementary Data 9 and Supplementary Figure 5) biosynthesis/signaling genes. The analysis revealed that numerous SA-related genes were strongly upregulated by photoperiod stress (Figure 6). Transcriptional regulation of selected SA biosynthesis and signaling genes has been confirmed by qRT-PCR for *EDS1*, *PAD4, ICS1* and *PATHOGENESIS-RELATED GENE 1* (*PR1*) in WT and the stress-sensitive genotypes *ahk2 ahk3* and *cca1 lhy* (Figures 6B–E). Transcript levels of JA biosynthesis and signaling genes such as *LIPOXYGENASE3* (*LOX3*), *LOX4* and *JASMONATE-ZIM-DOMAIN PROTEIN 1* (*JAZ1*), were strongly upregulated as well after photoperiod stress in WT and the photoperiod stress-sensitive mutants (Supplementary Figure 5 and Supplementary Data 9). Besides the increased transcriptional regulation of SA and JA biosynthesis genes, also the levels of SA and JA-Ile were strongly increased after photoperiod stress in WT and the photoperiod stress-sensitive mutants (Figure 7).

**FIGURE 8:**
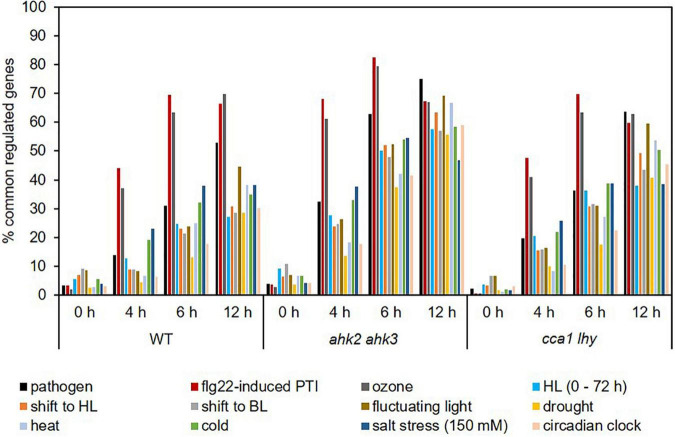
Photoperiod stress activates the plant pathogen response improving resistance against *Pseudomonas* infection. Five-week-old short-day (SD)-grown plants (WT, *ahk2 ahk3* and *cca1 lhy)* were exposed to a prolonged light period (PLP) of 32 h followed by a normal SD night. **(A)** Camalexin concentration at 15 h after photoperiod stress treatment. Error bars represent SE (*n* = 8). **(B,C)** Relative expression of *PAD3* **(B)** and *FMO1* **(C)** in WT, *ahk2 ahk3* and *cca1 lhy* plants during the night following the 32 h PLP measured by qRT-PCR. Only results for PLP-treatment are presented. All values are expressed relative to 0 h WT control which was set to 1. Error bars represent SE (*n* ≥ 3). **(D,E)** Bacterial growth in *Arabidopsis* WT plants pretreated with a 32 h **(D)** or 8 h **(E)** PLP. **(F)** Bacterial growth in photoperiod stress-sensitive mutants pretreated with 16 h PLP. Bacterial infection with *Pseudomonas syringae pv. tomato* DC3000 (inoculum OD_600_ = 0.002) occurred during the day following the PLP and bacteria were extracted from leaves 3 days later. Error bars represent SE (*n* = 8). Letters indicate different statistical groups (*p* ≤ 0.05) as determined by a Welch *t*-test **(D,E)** or an ANOVA followed by Tukey’s *post-hoc* test **(A–C,F)**.

During pathogen defense responses, SA is an essential signaling molecule for both basal defense mechanisms and SAR. In addition, SA induces the formation of camalexin, which is one of the major phytoalexins in plant defense responses decreasing bacterial growth after an infection (Glawischnig, 2007). After photoperiod stress, camalexin concentrations increase strongly and the transcript levels of *PHYTOALEXIN DEFICIENT3* (*PAD3*), a key enzyme of camalexin biosynthesis increased upon photoperiod stress treatment (Figures 8A,B). In addition to these SA-dependent defense responses, we also observed that transcript levels of *FMO1*, which encodes flavin-dependent mono-oxygenase that is an essential component of the biologically induced SAR independent of SA (Mishina and Zeier, 2006), is strongly upregulated during the night at 6 and 12 h after photoperiod stress treatment (Figure 8C). Together, these results clearly demonstrate that photoperiod stress induces responses similar as pathogen infection.

### Photoperiod Stress Pretreatment Improves Plant Pathogen Defense Response

Because plants respond to photoperiod stress similar as to pathogen infection we asked whether photoperiod stress enhanced the plant resistance against (hemi-) biotrophic pathogens.

To answer this question, we inoculated previously photoperiod-stressed and none-stressed plants with *Pseudomonas syringae* pv. *tomato* DC3000 (*Pst*). In the first experiment, we inoculated not yet fully developed leaves (11–13) with *Pst* in the morning the day after the photoperiod stress treatment (24 h light prolongation). These leaves were chosen as they do not become flabby as mature leaves do after a 24 h prolonged light period (Nitschke et al., 2016), which might interfere with the pathogen infection. Colony forming units were counted 3 days post infection. In plants pretreated with photoperiod stress, bacterial growth was strongly reduced (Figure 8D).

In a second experiment, we inoculated mature leaves but decreased the duration of the photoperiod stress treatment to 8 h light prolongation (16 h PLP) thus avoiding the flabby phenotype in mature leaves. This pretreatment also decreased the bacterial growth in WT (Figure 8E). Similar trends were observed in the photoperiod stress-sensitive genotypes (Figure 8F).

The results of both experiments demonstrate that photoperiod stress results in an enhanced immunity in the absence of a pathogen.

## Discussion

In this study, we have analyzed the transcriptomic changes in response to photoperiod stress. A prolongation of the light period by 24 h resulted in massive transcriptomic changes during the night following the extended light period in WT *Arabidopsis* plants and even stronger changes in the photoperiod stress-sensitive *ahk2 ahk3* and *cca1 lhy* mutants (Figure 1). The first transcriptional changes precede the development of the first visible photoperiod stress symptoms (Nitschke et al., 2016). The steadily increasing number of DEGs during the night reflects the appearance and progression of the physiological photoperiod stress phenotype. Even more, the stronger transcriptomic changes in the *ahk2 ahk3* and *cca1 lhy* mutants reflect their higher photoperiod stress sensitivity, which becomes apparent in their stronger physiological responses like increased oxidative burst, stronger reduction of maximum quantum efficiency and more lesion formation (Nitschke et al., 2016; Abuelsoud et al., 2020).

One of the characteristics of photoperiod stress is the nightly increase of oxidative stress, accompanied by the formation of peroxides (Abuelsoud et al., 2020). Therefore, as expected, among the top 20 DEGs and top 5 GO terms are genes related to oxidative stress and GO terms related to stresses causing an oxidative burst, respectively (Figures 1–3 and Supplementary Tables 6–12). Several of these DEGs (e.g., *OXI1*, *RBOHC*, *PRX4* and *PRX37*) are known to be involved in the regulation of an oxidative burst after biotic or abiotic stresses (Rentel et al., 2004; Valerio et al., 2004; Daudi et al., 2012; O’Brien et al., 2012; Kaya et al., 2019) or to be transcription factor genes (e.g., *ZAT11* and *ERF71*) responsive to H_2_O_2_ (Hieno et al., 2019). Comparison of the photoperiod stress-responsive genes with the list of ROS core-set genes, which is based on the meta-analysis of several transcriptomic studies, revealed a strong regulation of these genes (Figure 4 and Supplementary Data 4). Consistent with a prominent role of ROS in the photoperiod stress response, the stress induced a remarkable transcriptional deregulation of genes coding for enzymes involved in the scavenging of ROS including GSTs (Figure 4 and Supplementary Figure 4). In accordance, the activities of ROS scavenging enzymes, especially of catalases and peroxidases, were strongly altered after photoperiod stress (Abuelsoud et al., 2020). Taken together, these results indicate that ROS, which are known to act as signaling molecules and as transcriptional activators (Vaahtera et al., 2014; Willems et al., 2016), are important for the photoperiod stress response.

Comparison of the transcript profile in response to photoperiod stress to those in response to other biotic and abiotic stresses revealed a strong overlap with transcriptional regulation by ozone, pathogen attack and flg22-induced PTI (Figure 5 and Supplementary Data 7). Common to these stresses is the occurrence of an apoplastic oxidative burst that occurs also during the night following the photoperiod stress (Bolwell et al., 1999; Torres and Dangl, 2005; Van Breusegem and Dat, 2006; Abuelsoud et al., 2020).

An apoplastic oxidative burst triggers SA signaling during PTI and ETI defense responses (Bolwell et al., 2002) and during plant responses to ozone and UV-B (Herrera-Vasquez et al., 2015). In a feed-forward loop, SA promotes ROS production by the inhibition of catalase and ascorbate peroxidases during plant defense responses (Chen et al., 1993) and by the stimulation of extracellular peroxidases in stomata during drought stress (Khokon et al., 2011). However, SA can also promote ROS scavenging to limit the oxidative burst, e.g., during ozone stress (Yoshida et al., 2009), and in responses to avirulent bacteria (Grant and Loake, 2000). In addition, SA can modulate glutathione levels (Mateo et al., 2006). Thus, a complex interaction network exists between the apoplastic oxidative burst, SA and defense responses.

During photoperiod stress, genes involved in SA biosynthesis and signaling are consistently upregulated (Figure 6 and Supplementary Data 8). As the increase in SA levels occurs only transiently at the end of the night and ROS levels decrease again during the following day (Abuelsoud et al., 2020), it might be that SA stimulates ROS scavenging.

Besides the increased SA levels, also JA (Figure 7 and Supplementary Figure 5) and camalexin (Figure 8) levels are strongly induced. Both play an important role in resistance against necrotrophic pathogens (Kliebenstein et al., 2005; Spoel et al., 2007; Van Baarlen et al., 2007). Together, our results indicate plant responses to photoperiod stress are highly similar to responses to phytopathogens.

The surprising similarity between an abiotic stress (photoperiod stress) and biotic stress prompted us to explore whether photoperiod stress has an impact on the plant defense response against pathogens. Because a first pathogen infection improves responses to future pathogen attacks (Conrath et al., 2001), we investigated whether also photoperiod stress improves plant pathogen resistance. Indeed, photoperiod stress pretreatment decreased bacterial growth after *Pseudomonas* infection (Figure 8). As photoperiod stress is associated with increased SA, JA and CK levels (Nitschke et al., 2016; Frank et al., 2020), the improved pathogen resistance might be mediated by these hormones and their downstream signaling pathways. While SA and CK activate resistance mechanisms against biotrophic and hemi-biotrophic pathogens, JA is crucial for the activation of defense responses against necrotrophic pathogens (Bari and Jones, 2009; Cortleven et al., 2019).

CK is known to potentiate SA-dependent defense responses via the AHK2/AHK3 receptors and via the interaction of ARR2 with TGA3 and NONEXPRESSOR OF PATHOGENESIS-RELATED GENE1 (NPR1) to induce post-invasive defense mechanisms (Choi et al., 2010). As a consequence, the photoperiod stress-sensitive mutant *ahk2 ahk3* is also more sensitive to pathogen infection (Choi et al., 2010). This is in accordance to the results in our study (Figure 8F). However, photoperiod stress pretreatment improved the resistance to *Pst* infection also in *ahk2 ahk3* mutants indicating that photoperiod stress primes plant immunity independent of the CK signaling pathway. An increase of pathogen resistance after photoperiod stress in *Arabidopsis* was not only observed after *Pseudomonas* infection (this study) but also after infection with the hemi-biotrophic fungus *P. oryzae* (syn. *M. oryzae*) (Shimizu et al., 2021) and after infection by the necrotrophic fungus *B. cinerea* (Cagnola et al., 2018). In these studies short day-grown plants were transferred to long day conditions just before infection, which corresponds to a photoperiod stress treatment and resulted in an increased resistance against *P. oryzae* and *B. cinerea*. Further research is necessary to unravel the signaling pathways contributing to enhanced resistance to biotrophic, hemi-biotrophic, and necrotrophic pathogens by photoperiod stress.

Notably, a prolonged light treatment enhances pathogen resistance not only in *Arabidopsis*, but also in tomato plants. A nightly red light treatment improves the resistance against *P. syringae* pv. *tomato* DC3000 (Yang et al., 2015). This treatment enhanced the SA level and expression of SA signaling genes, and expression of genes involved in redox homeostasis like *RBOH* and *GSTs* was strongly altered, which is in accordance to our results (Yang et al., 2015). This study demonstrated that the photoperiod stress response is not limited to *Arabidopsis* but is more widely distributed provoking similar responses in different plant species.

The fact that photoperiod stress improves pathogen resistance might be exploited to enhance plant defense responses. It remains an open question, how the pre-treatment of plants with photoperiod stress improves resistance against pathogens. Our data suggest that photoperiod-stress mediated higher resistance is associated with increased ROS levels after photoperiod stress. In addition other known immune pathways based on SA/JA signaling might be involved. Further research is necessary to unravel the underlying mechanisms.

## Data Availability Statement

The datasets presented in this study can be found in online repositories. The names of the repository/repositories and accession number(s) can be found below: https://www.ncbi.nlm.nih.gov/geo/, GSE173899.

## Author Contributions

AC and TS developed and coordinated the project, and wrote the article with contributions of all other authors. AC, MF, VR, and VL performed the experiments. AC, MF, VR, VL, and TS analyzed the data. AC, JB, and GB performed statistical analysis of the RNA-seq data. All authors contributed to the article and approved the submitted version.

## Conflict of Interest

The authors declare that the research was conducted in the absence of any commercial or financial relationships that could be construed as a potential conflict of interest.

## Publisher’s Note

All claims expressed in this article are solely those of the authors and do not necessarily represent those of their affiliated organizations, or those of the publisher, the editors and the reviewers. Any product that may be evaluated in this article, or claim that may be made by its manufacturer, is not guaranteed or endorsed by the publisher.

## Supplementary Material

Supplementary Figure 1 General overview of the RNA-seq dataset. **(A)** PCA of the biological samples. Control samples cluster together (red circle). A strong diversification of light-treated samples is visible with a strong separation of the different genotypes at time point 12 h (blue circles). **(B)** Venn diagram showing the overlap of treatment, genotype and time—dependent DEGs. Analysis is based on the outcome of DEseq2 analysis (three-factor analysis). **(C,D)** QT clustering of differentially expressed genotype-dependent genes. 52% of all significantly regulated genotype-dependent genes belong to cluster 1 **(C)** and cluster 2 **(D)**. An overview of the other clusters can be found in Supplementary Figure 2. **(E,F)** GO term analysis of genes belonging to cluster 1 **(C)** and to cluster 2 **(D)**.

Supplementary Figure 2 QT clusters of significantly regulated genotype-dependent genes. QT clustering of differentially expressed genotype-dependent genes after 32 h photoperiod stress in WT, *ahk2 ahk3* and *cca1 lhy* mutants.

Supplementary Figure 3 Overlap of photoperiod stress responsive transcription factor genes with different datasets describing ROS-responsive transcription factor genes. **(A)** Venn diagram showing the overlap between the ROS-responsive genes identified by Gadjev et al. (2006), Lai et al. (2012), Hieno et al. (2019) and Zandalinas et al. (2019). Number in brackets are the total numbers of genes found in the respective study. **(B)** Venn diagrams showing the proportion of the 283 ROS-responsive transcription factor genes (TF) shown in **(A)** that are induced or repressed by photoperiod stress in the different genotypes at different time points. **(C)** Percentage of DEGs induced by photoperiod stress co-regulated with genes responsive to H_2_O_2_, superoxide and singlet oxygen. An overview of the regulation of these transcripts after photoperiod stress is provided in Supplementary Data 3, 4. WT, wild type.

Supplementary Figure 4 Photoperiod stress causes a strong transcriptional regulation of genes coding for enzymes involved in the scavenging of ROS. 5-week-old short-day (SD)-grown plants were exposed to a prolonged light period (PLP) of 32 h followed by a normal SD night. The experimental setup is shown in Figure 1A. **(A)** QT clustering of genes encoding enzymes involved in the scavenging of ROS (Pearson’s correlation). Three clusters are found: cluster 1 showing upregulation of genes over time, cluster 2 showing downregulation of genes over time and cluster 3 showing a strongly increased expression at time points 4 and 6 h. Depicted are the ratios of PLP vs. control plants for each genotype and time point. A complete list of the genes coding for scavenging enzymes used in this analysis including their expression levels is provided in Supplementary Data 6.

Supplementary Figure 5 Photoperiod stress results in transcriptional regulation of jasmonic acid biosynthesis and signaling. Hierarchical clustering of genes involved in jasmonic acid (JA) biosynthesis and signaling (Pearson’s correlation). An overview of the regulation of these JA biosynthesis and signaling genes after photoperiod stress treatment is provided in Supplementary Data 9.
