# Supplementary figures and images for: Photoperiod Stress in Arabidopsis thaliana Induces a Transcriptional Response Resembling That of Pathogen Infection
Source: Front Plant Sci. 2022 May 12;13:838284. doi: 10.3389/fpls.2022.838284 (PMC9134115; doi:10.3389/fpls.2022.838284)

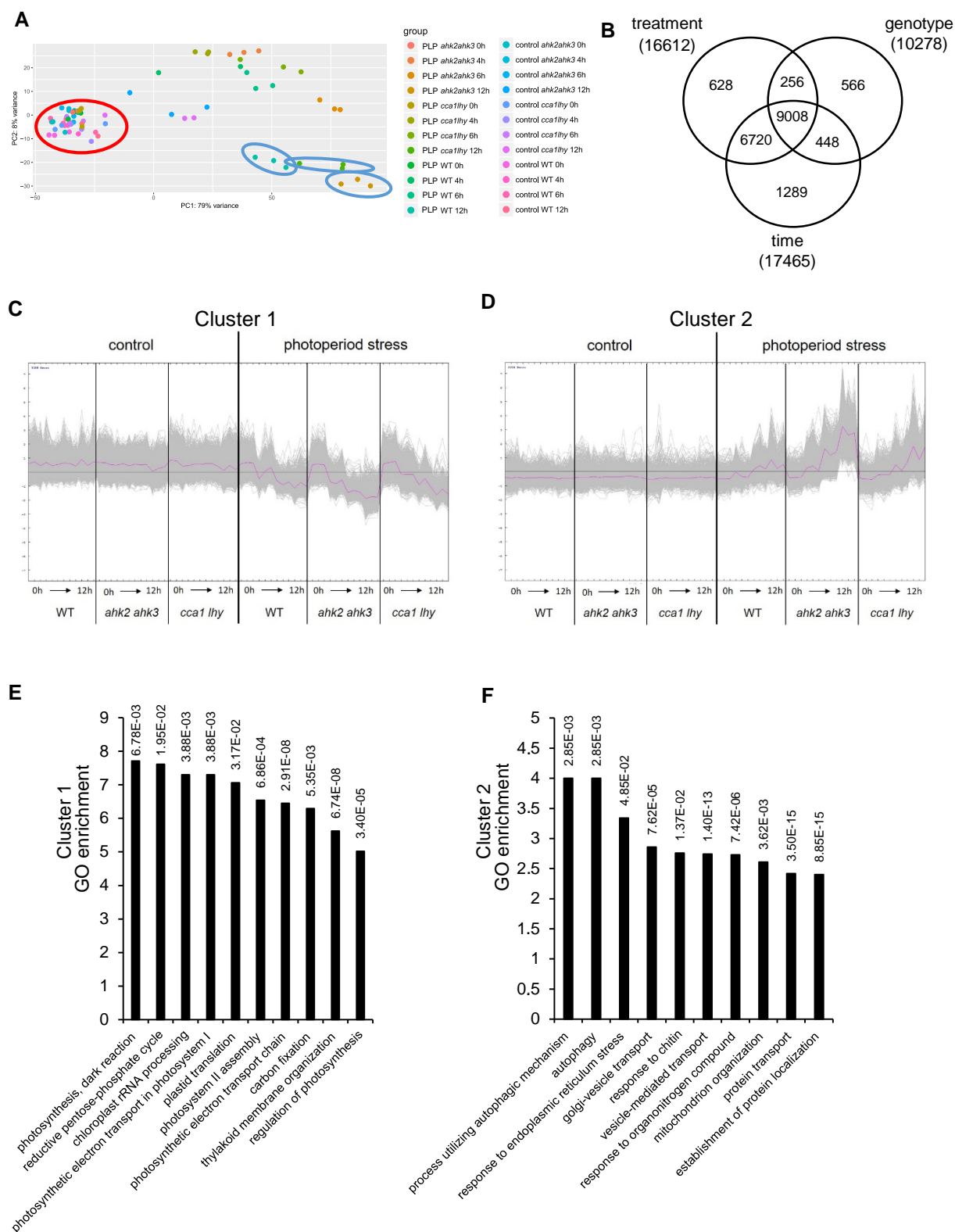

Supplementary Figure 1



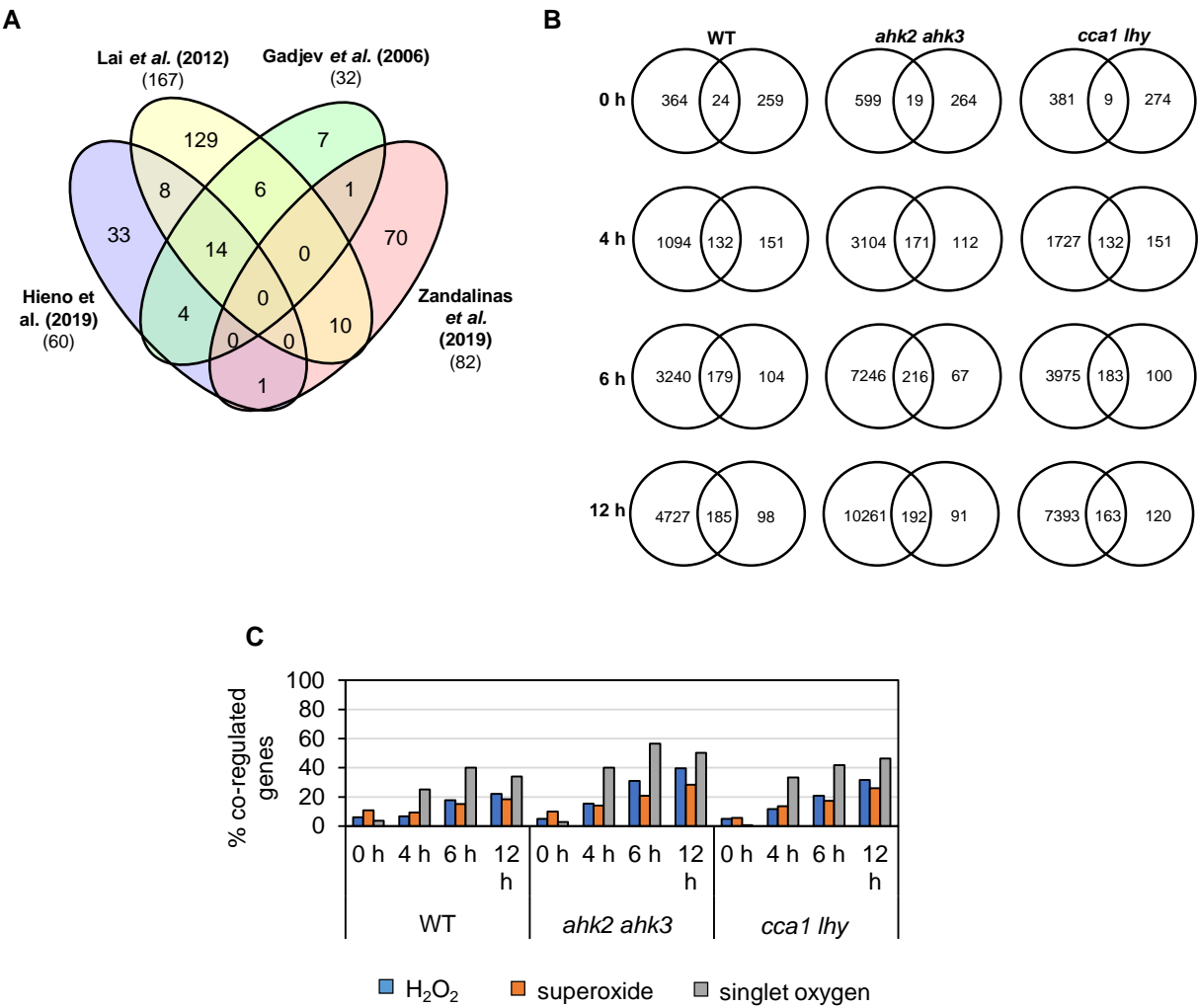

Supplementary Figure 3

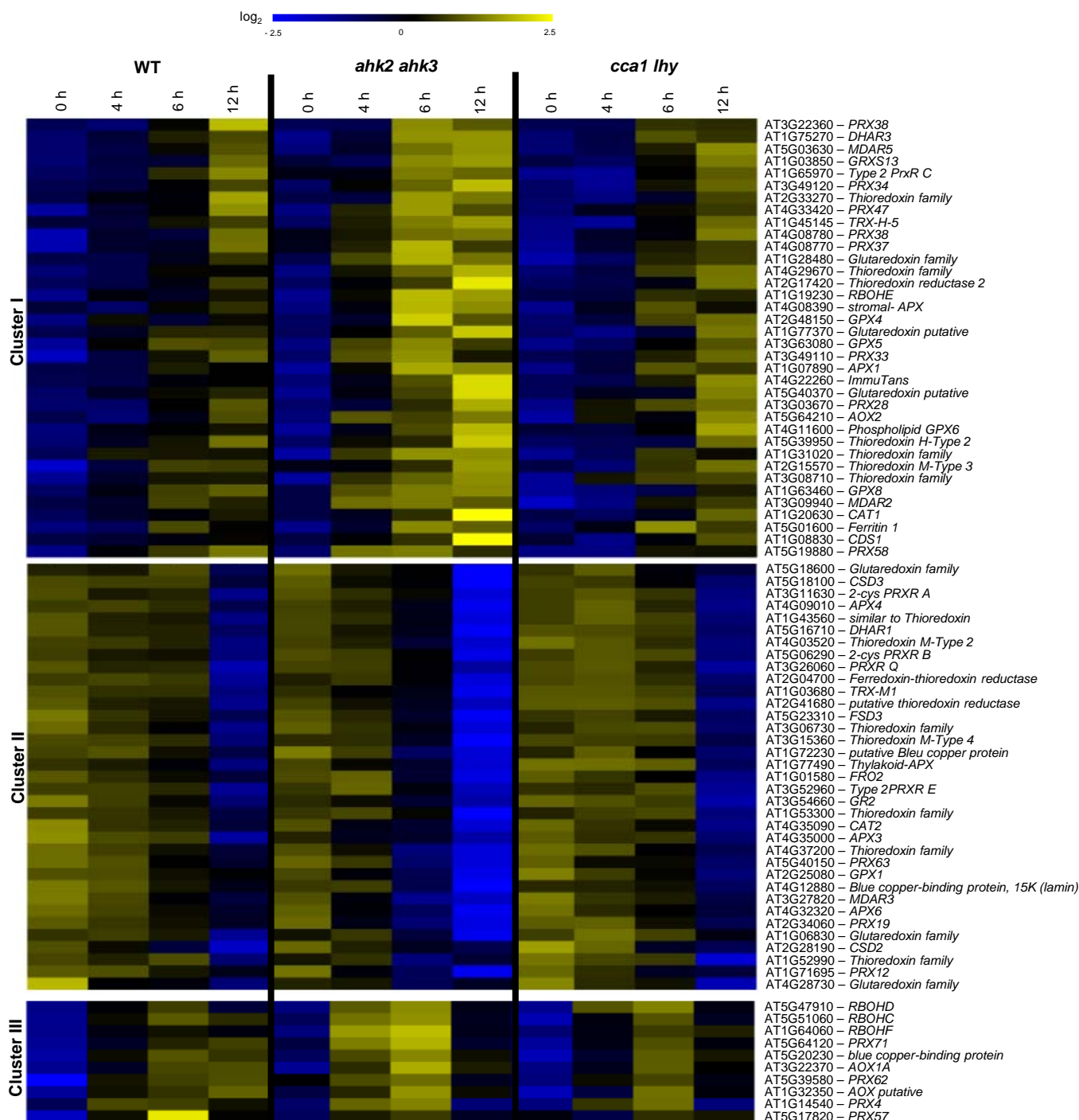

Supplementary Figure 4

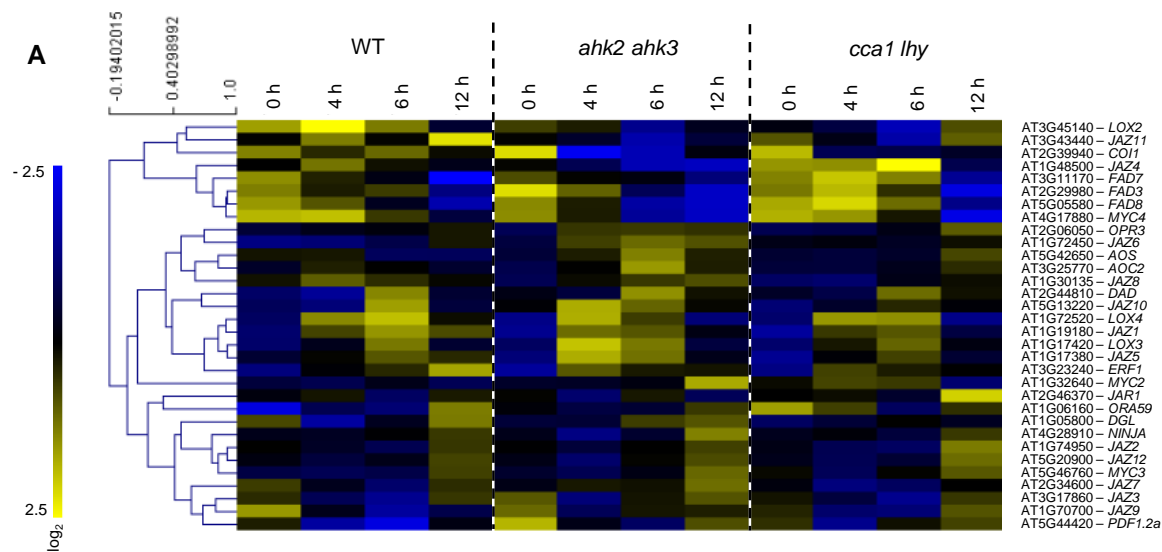

Supplementary Figure 5

Supplement: Supplementary file 1 [file Data_Sheet_1.zip › DataSheet/Image 1.pdf]
